# Perception of Ophthalmologists of COVID-19 Using the Health Belief Model

**DOI:** 10.7759/cureus.12681

**Published:** 2021-01-13

**Authors:** Enmar M Almazyad, Abeer Ahmad, Deema E Jomar, Rajiv B Khandekar, Samar Al-Swailem

**Affiliations:** 1 Ophthalmology/Research Department, King Khaled Eye Specialist Hospital, Riyadh, SAU; 2 Research Department, King Khaled Eye Specialist Hospital, Riyadh, SAU; 3 Ophthalmology Department, King Khaled Eye Specialist Hospital, Riyadh, SAU; 4 Department of Ophthalmology, Faculty of Medicine, University of British Columbia, Vancouver, CAN

**Keywords:** covid-19, health measures, ophthalmology, health belief model

## Abstract

Purpose

To assess ophthalmologists' preparedness in such a critical period in the history of pandemics, a logical socio-psychological framework assessment using the health belief model (HBM) is essential to evaluate their risk perception, their willingness to actively participate in engaging in protective health behavior and acknowledge its benefits, and their capability to perform adequate successful methods for limiting the spread of coronavirus disease 2019 (COVID-19) and overcome the barriers they might encounter while implementing such precautions.

Methods

A cross-sectional study conducted at King Khaled Eye Specialist Hospital using a questionnaire-based (HBM) was distributed to 135 ophthalmologists in the institute to evaluate their risk perception of COVID-19 and determine which components of the HBM contribute to preventive health behavior related to the COVID-19 infection.

Results

The questionnaire had a reasonable response rate (79.3%, 107 ophthalmologists, including 48 consultants, 51 fellows, and 36 residents). The study demonstrated that this model is useful and mapped how several components were significantly correlated to actions. Most significantly, perceived susceptibility was the most important predictor of action. The second most important determinant of action was the perceived benefit.

Conclusion

Pandemics such as COVID-19 are likely to happen again in the future. Explicit attention to factors influencing motivation such as threat perception to adopt appropriate health-related behavior to limit the spread of communicable diseases is necessary. This study has successfully represented preparedness and risk behavior perception of ophthalmologists of the novel COVID-19 pandemic in one of the largest tertiary eye hospitals in the Middle East using the Health Belief Model.

## Introduction

Coronavirus disease 2019 (COVID-19) is a current international pandemic emergency declared by the World Health Organization (WHO) on March 11, 2020 [1]. Since then, it has had a significant impact on the economy, society, and, most importantly, medical practice worldwide, including ophthalmology [2].

As there is no specific treatment for COVID-19 and vaccines are still under clinical trials, the current approach is directed toward alleviating the virus’s signs and symptoms, as well as limiting the spread of the outbreak by global WHO efforts and local governmental public health policies [3-4].

Since the outbreak, healthcare workers worldwide, ophthalmologists in particular due to the nature of their work and closeness to patients during the examination, are concerned about their exposure to this fast-spreading virus [5-6]. Moreover, many publications have emerged suggesting that the virus can present as conjunctivitis and its possibility of transmission through ocular tissue [7-10]. In addition, several ophthalmic societal groups are in consensus about the risk of this novel virus. Consequently, they have issued strategical plans to provide optimal ophthalmic care while ensuring ophthalmologists' personal and work-field safety by raising their awareness and educating them to practice appropriate precautions [11].

However, whether ophthalmologists will trust and be willing to comply with recommended precautionary behaviors is not absolute. Researchers have developed several social-psychological frameworks to assess willingness to engage (or lack of engagement) in preventive measures and predict health-related behaviors. The most well-known and favorably influential is the health belief model (HBM) [12-14]. The HBM has been used to develop appropriate interventions to change health-related behaviors by targeting various aspects of the model's key constructs and was extremely useful in previously recognized communicable diseases [15-20].

According to the HBM, modifying factors, such as perceptions of the disease, perceptions of behavior, and cues to action, simultaneously influence the likelihood of taking a recommended preventive health action. The value of health-related behaviors is avoiding sickness. The expectation is that a specific health action could prevent the condition for which people consider they might be at risk [13-14,21-23].

In order to assess ophthalmologists preparedness and behavior in such a critical period in the history of pandemics, a logical socio-psychological framework assessment using HBM is essential to evaluate their risk perception, their willingness to actively participate in engaging in protective health behavior and acknowledge its benefits and their capability to perform adequate successful methods in limiting the spread of COVID-19 and overcome the barriers they might encounter while implementing such precautions. Therefore, the present study aims to evaluate ophthalmologists risk perception of COVID-19 in one of the largest tertiary eye hospitals in the Middle East and Gulf region, King Khaled Eye Specialists Hospital, using the Health Belief Model (HBM) and determine which components of the HBM contribute to preventive health behavior related to the COVID-19 infection.

## Materials and methods

Study design

The study is based on a cross-sectional, web-based questionnaire conducted at King Khaled Eye Specialist Hospital, Riyadh, Saudi Arabia, during the beginning of the COVID-19 pandemic (April-May 2020). The target population comprises ophthalmologists at different levels (residents, fellows, and consultants). The King Khaled Eye Specialist Hospital institutional review board approved the study (Project approval reference number: 2044). Informed consent was implemented at the beginning of the web-based questionnaire to explain the purpose of the study. Participation was voluntary, and ophthalmologists could withdraw their participation at any time.

Questionnaire

The questionnaire consisted of the following parts: (a) items requesting sociodemographic information, including age, gender, marital status (single, married, widowed), living status (living alone, living with family members or with spouse and kids), nationality, experience/level at work (resident, fellow, consultant), subjected to quarantine (yes, no), the reason of quarantine (travel, exposure); (b) health status: any chronic diseases (yes, no), what are they (diabetes mellitus, asthma, other), for women (pregnant or not); (c) items that are measuring HBM variables, including the four categories of susceptibility, severity, benefits, and barriers, self-efficacy, and cues to action (the items in each category are shown in Appendix 1).

For the different components, multiple questions were included. We formulated these questions using other referenced studies that were aiming to explore different determinants for engaging in certain health behavior [15-20,24]. The questions were also formulated in an agreement between epidemiologists and researchers in the institute's research department.

Items in the HBM predictor categories were measured on a five-point Likert-type scale, with the following possible responses: strongly disagree (1), disagree (2), neither agree nor disagree (3), agree (4), and strongly agree (5). Each of the HBM scales was a sum of answers for several questions. The scores on each of the scales were averaged to form the independent variable higher scores indicating higher risk perception, perceived severity, benefits, barriers, and self-efficacy.

Data analysis and sample size

Our primary outcome measure is to find the relation between different components and compliance with preventive behavior (action). To obtain a sample size, we have focused on an essential determinant of engaging in a preventive behavior such as perceived susceptibility. We hypothesize that there is no correlation between the two components (r=0). However, we assume that on finding a moderate positive correlation between perceived susceptibility and preventive behavior (action) of approximately 0.6, with 80% of power (adequate power in psychology research) and an alpha of 5%, we will need around 56 ophthalmologists (Statistical software PASS Version 2020; Power Analysis and Sample Size Software (2020). NCSS, LLC. Kaysville, Utah).To compensate for non-responders, we included an additional 20%, resulting in a total of 67.

Item analysis was performed for each component to measure the reliability using Cronbach's alpha. Questions that increased the reliability of components were removed to eliminate overestimation. For items with a low Cronbach's alpha, the results should be reported carefully. To measure the association of the different components of the HBM model, with the "action" component scores, a Pearson correlation coefficient will be calculated. A multiple regression analysis was performed to analyze to what extent the different components of the HBM model determine the dependent variable "Action" (preventive measures). The regression coefficient, 95% confidence intervals, will be presented. Significance for the correlation will be set at a p-value of <0.05.

## Results

Out of 135 ophthalmologists in King Khaled Eye Specialist Hospital, 107 responded to the questionnaire, with a 79.3% response rate, including 48 consultants, 51 fellows, and 36 residents. Table 1 summarizes the participants’ characteristics and demographics.

**Table 1:** Ophthalmologist (N=107) demographics and characteristics

Ophthalmologist demographics and characteristics		
Characteristics	Number	Percentage%
Gender		
Male	63	58.9
Female	44	41.1
Nationality		
Saudi	94	87.9
Non-Saudi	13	12.1
Age (Mean±SD)	34.39±9.725	
Minimum	25	
Maximum	63	
Marital status		
Married	46	43
Single	61	57
Pregnancy status of females		
Pregnant	4	9.1
Not pregnant	40	91.9
Living status		
With family	80	74.8
Alone	27	25.2
Experience level		
Consultant	35	32.7
Fellow	40	37.4
Resident	32	29.9
Experienced quarantine		
Yes	16	14.9
No	91	85.1
Reason for quarantine (if quarantined)		
Recent travel	11	68.8
Early COVID-19 symptoms	2	12.5
Not known	3	18.7
Comorbidities		
Diabetes	4	3.7
Hypertension	6	5.6
Asthma	8	7.5
Dyslipidemia	2	1.7
Ischemic heart disease	1	0.9
Non-Ischemic heart disease	1	0.9
SLE	1	0.9
None	89	83.2
Combination	4	3.7

Cronbach’s alpha shows a reliable unidimensional module per each HBM component (Table 2). All components had a high average mean score on the Likert scale except for perceived barriers (Table 3).

**Table 2:** Cronbach’s alpha per HBM component HBM: health belief model

Cronbach’s alpha per HBM component	
Components	Cronbach's alpha
Perceived Susceptibility	0.78
Perceived Severity	0.69
Perceived Benefits	0.73
Perceived Barriers	0.76
Self-efficacy	0.71
Cues to action	0.67
Action	0.79

**Table 3:** Mean component HBM model (N=107) HBM: health belief model

Mean component HBM model (N=107)		
Components	Mean score	SD
Perceived Susceptibility	4.3	0.41
Perceived Severity	4.1	0.55
Perceived Benefits	4.1	0.68
Perceived Barriers	2.7	0.80
Self-efficacy	3.7	0.68
Cues to Action	4.0	0.58
Action	4.6	0.40
The average score on the questionnaire is shown per the health belief model component, with the following distribution: strongly disagree agree (1), disagree (2), neither (3), agree (4), and strongly agree (5).		

**Figure 1:**
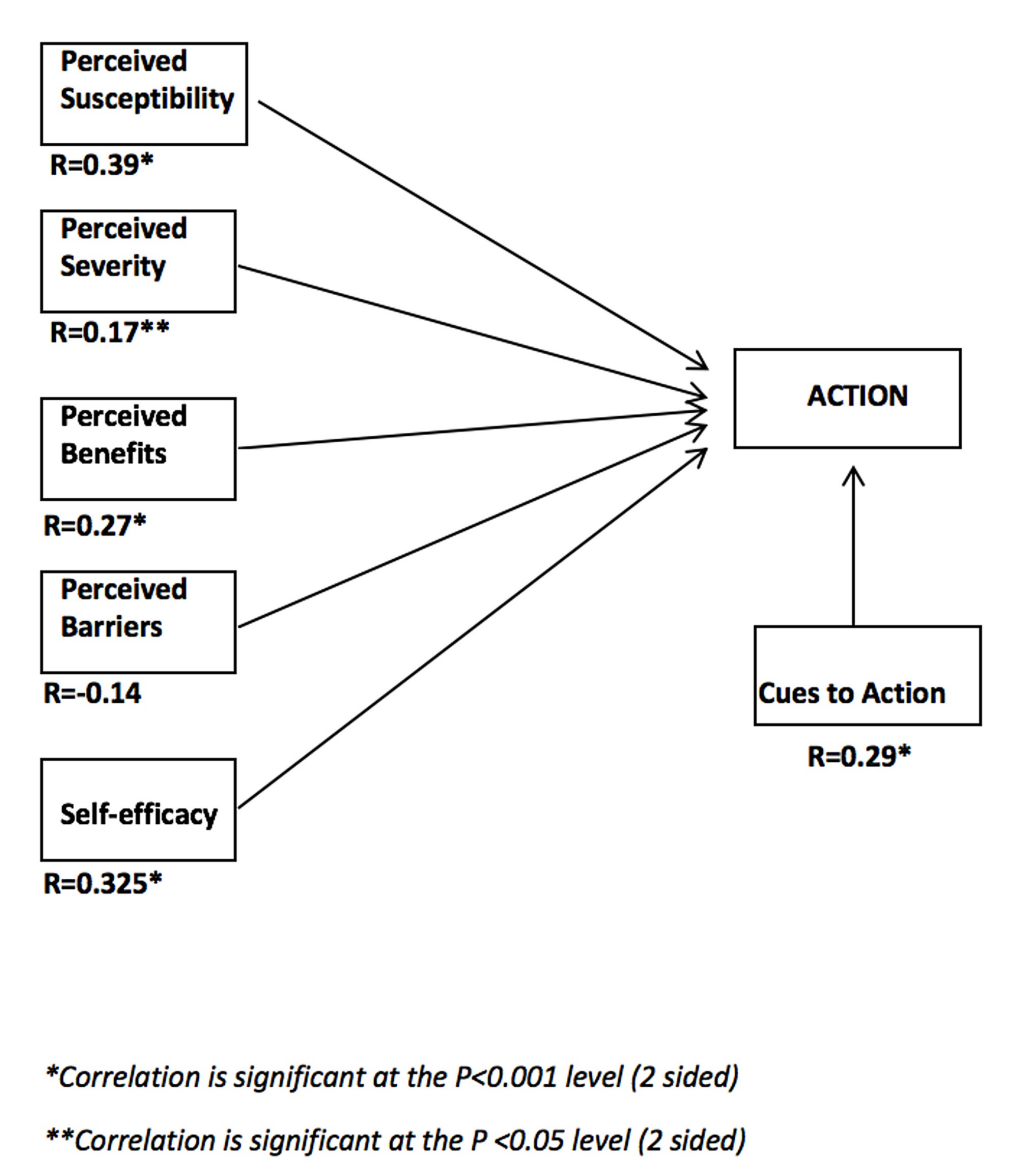
Correlation between the different components of the health belief model (HBM) and action

Figure 1 illustrates the correlation between the different components of the HBM and action. The components, perceived susceptibility, perceived severity, perceived benefits, self-efficacy, and cues to action all show a significant positive relation with action. Perceived susceptibility has the highest correlation coefficient and is, therefore, most closely related to action. This positive correlation shows that the higher ophthalmologists perceive their susceptibility to the disease, the more they carry out protective behavior. This is also the case for perceived severity, perceived benefits, self-efficacy, and cues to action. There was no significant correlation perceived between perceived barriers and actions. However, there is a negative correlation, meaning the lower the perceived barriers, the higher their compliance with protective behavior and vice versa (Figure 1).

Additionally, multiple regression analysis was performed to see to what extent the different components of the HBM explained the dependent variable action. The regression coefficient (B) was adjusted for confounding factors such as age, gender, marital status, living status, subjected to quarantine, and systemic comorbidities. Overall, all components together (perceived susceptibility, perceived severity, perceived benefit, perceived barriers, self-efficacy, and cues to action) accounted for 65% of the variance in action. The regression analysis demonstrated that action could be explained significantly by the components of the HBM (F=5.788, p<0.001). Most importantly, perceived susceptibility and perceived benefit had significantly affected action. In fact, according to the regression analysis, perceived susceptibility could be considered the best predictor of action, as it had the most significant effect on the action (β = 0.378, p<0.001) (Table 4).

**Table 4:** Multiple regression analysis of the relation between HBM components and action (N=107) HBM: health belief model

Multiple regression analysis of the relation between HBM components and action		
Components	Beta (B)*	Significance (P)
Perceived susceptibility	0.378	0.001**
Perceived severity	0.072	0.261
Perceived benefit	0.156	0.004**
Perceived barriers	-0.062	0.185
Self-efficacy	0.092	0.087
Cues to action	0.102	0.083
Variance	0.65	0.001
*Regression coefficient (B) adjusted for age, gender, marital status, living status, subject to quarantine, and systemic co-morbidities. ** Significant p<0.05		

## Discussion

This study has successfully represented the preparedness and risk behavior perception of ophthalmologists of the novel COVID-19 pandemic in one of the largest tertiary eye hospitals in the Middle East using the HBM. After an extensive literature review using search engines PubMed, Google Scholar, Researchgate, and Cochrane, this is the first reported study that has constructed a focused, mapped analysis that described ophthalmologists’ perception behavior to the current COVID-19 pandemic.

This model mainly emphasizes the attitudes and beliefs of individuals to explain and predict their health behavior. It primarily focuses on two main aspects of health behavior, threat perception and behavioral evaluation. Threat perception consists of the disease's perceived susceptibility and the perceived severity of the consequences of the disease, and it plays an essential role in health-related behavior. The behavioral evaluation consists of the perceived benefits of a recommended health behavior and possible barriers that individuals may encounter to engage in this health behavior. It also incorporates two critical elements into its assessments about what it takes to get an individual to be more engaged in such behaviors. These two elements are cues to action and self-efficacy [13-14,21-22,25-26].

We learned from previous outbreaks, such as influenzas in 2003 and Ebola in 2013 [15,17,19], that the effectiveness of the control of communicable diseases epidemics is primarily determined by the perception of risk of the infection, health-related behavior of the population, and health care providers and their willingness to comply to recommended preventive methods. Therefore, to promote sufficient precautionary behavior among the population and health care providers, public health authorities need to know how individuals perceive risks and their severity, how they perceive the effectiveness and acknowledge precautionary methods such as hygiene, quarantines, wearing masks, maintaining social distance and following patients' care protocols by physicians [13-16,18-27].

Likewise, O Zawart’s thesis divided large-scale comparative studies into perceived threat and risk perception of emerging infectious diseases, including severe acute respiratory syndrome (SARS) and avian influenza. He concluded that most studies on SARS and avian influenza found an association between higher risk perception and engaging in precautionary actions. Thus, suggesting that in stimulating precautionary actions in the control of outbreaks, specific attention should be paid to stimulate a high enough perceived threat to ensure that people will engage in these precautionary actions and enhance their efficacy beliefs and their trust in doing so [17-19]. Similarly, this current study mapped ophthalmologists' behavior utilizing HBM, and it showed that perceived susceptibility, perceived severity, perceived benefits, self-efficacy, and cues to action all had a significant relation with action. In particular, perceived susceptibility has the highest correlation and is accordingly the most closely related to action. These correlations interpret that the higher ophthalmologists perceive their susceptibility for COVID-19, the more they carry out protective behavior.

Furthermore, multiple regression analysis revealed that perceived susceptibility was the best predictor of action and, therefore, had the most significant effect on action.

Also, we have to consider that threat perception is not the only determinant of protective behavior. Individuals have to believe that the benefit of a recommended health behavior is efficient and have the confidence of self-competence to apply successful behavior, which is described by the theory of response efficacy and self-efficacy, respectively. Our findings have confirmed this concept, as the perceived benefit was the second most crucial component of the model, influencing action [12-15,17-19,21-23].

Possibly, such findings are rationalized by the fact that ophthalmologists are conscious that they are at high risk of contracting COVID-19 and have substantial exposure among other healthcare workers due to their proximal working distance to patients along with extended critical clinical examination duration their practice and surgery. Consequently, affected their health risk behavior. One advantage of this study is that it took place at the beginning of the pandemic where the knowledge gap about the virus still existed, making it an accurate representation of ophthalmologists’ alertness in a time where evidence about a novel virus pandemic was still limited.

Nevertheless, whether these findings represent all ophthalmologists' perception worldwide of this pandemic as a response to global health care efforts or a reflection of local public health efforts that vary between institutes and countries these positive results still support previously mentioned socio-psychological study's outcomes. Emphasizing the importance of measuring these factors and address them adequately to achieve successful compliance and control of infectious disease pandemics [11-12,13-15,17-19,21-23].

Finally, our study limitation was related to the fact that most reviewed articles with HBM-based questionnaires lacked an existing validated questionnaire for perceived threat and risk perception of infectious diseases. Therefore, the questionnaire was specifically developed for the project reported in this paper (Appendix). It was based upon an earlier questionnaire used in previously discussed studies, then revised by two epidemiologists within our research department, and HBM component reliability was assessed by Cronbach’s alpha (Table 2).

## Conclusions

In conclusion, pandemics such as COVID-19 are more likely to happen more often in the future. Studies have found that an individual's desire to implement a change in health behavior is not enough to adhere to preventive measures and overcome barriers. Therefore, explicit attention to factors influencing motivation, such as threat perception to adopt appropriate health-related behavior to limit the spread of communicable disease, is necessary. To assess such behavior, the health belief model was used to give insight on risk perception and adhere to risk-reducing behavior. This study demonstrated that this model is useful and concluded how several components were significantly correlated to actions. Most significantly, perceived susceptibility was the most important predictor of action. The second most important determinant of action was the perceived benefit.

## Appendices

**Table 5:** Health belief model categories variables statements in the questionnaire WHO: World Health Organization; CDC: Centers for Disease Control and Prevention

Health belief model categories variables statements in the questionnaire	
Variables	Statements
Perceived Susceptibility	Working with many people each day increases my chances of getting the virus. If I have contact with patients, I increase my risk of contracting the virus. If I examine patients at a near distance without appropriate precautions I increase my chances of getting infected. If I examine non-emergent routine cases I’ll increase my chances of contracting the virus. If I delay and reschedule elective surgeries as well as clinic appointments of non-emergent ocular cases I’ll decrease my chances of contracting the virus. If I use a slit lamp barrier or breath shield, wash my hand frequently, wear gloves, and disinfect my instruments, I’ll decrease my chance of contracting the virus as well as its transmission to other patients. I believe that patient’s accumulation in the waiting area and lack of social distance increases the chance of virus transmission among them and my chance of getting the virus. I am concerned about getting infected with COVID-19 I have a feeling that COVID-19 is a big threat within this institute. I think I will be infected.
Perceived Severity	The virus is a serious disease. If I get infected with the virus, I can have fatal complications. If I get infected, it will affect my job and activities. If I contract the virus, I can transmit it to other patients as well as my family members. The thought of getting the virus makes me anxious.
Perceived Benefits	If I comply with health precautions (masks, washing hands, etc.), it will protect me and others from getting the virus. If I frequently disinfect my tools and use a slit lamp shield, it will protect me and others from getting the virus. If I cancel non-urgent cases in my clinics/OR, it will protect myself and others from getting the virus. If I would work from home, I would reduce my risk of getting infected.
Perceived Barriers	I find it difficult to comply with the recommended preventive measures. It takes too much time to apply precaution measures against infection. Preventive measures such as sanitary wipes, protective slit lamp shield, gloves, masks, and sanitary gels are not adequately provided by the institute/or vastly consumed. I can't cancel elective surgeries and appointments and reschedule them because it is not encouraged by the institute. It takes me too much time and effort to cancel elective surgeries and appointments and reschedule them.
Self-Efficacy	I am able to recognize any signs/symptoms of infection with the virus. I can do anything to protect myself against the virus. I know how to recognize the risks of contracting the virus. I can stay away from patients who have symptoms of illness.
Cues to Action	This institute provided me with enough information about the virus and preventive measures (posters, E-posters, emails). I have read enough information about COVID-19 in the newspaper, television, and social media. I have obtained information about COVID-19 through published scientific articles, the WHO website, and/or the CDC website. I have received enough information from my family and friends about the virus. The nurse frequently reminds me to take precautions ( wear my mask, wash hands, etc).
Actions	I will use my mask while at work. I will wash my hands regularly. I will use a slit lamp shield regularly. I will avoid touching my eyes, nose, and mouth with unwashed hands. I will cover my mouth when I am coughing and sneezing. I will keep my distance from possible sick people. I will disinfect my instruments regularly. I will cancel non-urgent appointments and surgery.
The five-point scale for the categories ranged from: Strongly disagree agree (1), disagree (2), neither (3), agree (4), and strongly agree (5).	
